# Genetic and Phenotypic Features to Screen for Putative Adherent-Invasive *Escherichia coli*

**DOI:** 10.3389/fmicb.2019.00108

**Published:** 2019-02-21

**Authors:** Carla Camprubí-Font, Christa Ewers, Mireia Lopez-Siles, Margarita Martinez-Medina

**Affiliations:** ^1^Laboratory of Molecular Microbiology, Department of Biology, Universitat de Girona, Girona, Spain; ^2^Institute of Hygiene and Infectious Diseases of Animals, Faculty of Veterinary Medicine, Justus-Liebig University Giessen, Giessen, Germany

**Keywords:** adherent-invasive *E. coli*, virulence genes, antibiotic resistance, molecular marker, virulence determinants

## Abstract

To date no molecular tools are available to identify the adherent-invasive *Escherichia coli* (AIEC) pathotype, which has been associated with Crohn’s disease and colonizes the intestine of different hosts. Current techniques based on phenotypic screening of isolates are extremely time-consuming. The aim of this work was to search for signature traits to assist in rapid AIEC identification. The occurrence of at least 54 virulence genes (VGs), the resistance to 30 antibiotics and the distribution of FimH and ChiA amino acid substitutions was studied in a collection of 48 AIEC and 56 non-AIEC isolated from the intestine of humans and animals. χ^2^ test was used to find frequency differences according to origin of isolation, AIEC phenotype and phylogroup. Mann–Whitney test was applied to test association with adhesion and invasion indices. Binary logistic regression was performed to search for variables of predictive value. Animal strains (*N* = 45) were enriched in 12 VGs while 7 VGs were more predominant in human strains (*N* = 59). The prevalence of 15 VGs was higher in AIEC (*N* = 49) than in non-AIEC (*N* = 56) strains, but only *pic* gene was still differentially distributed when analyzing human and animal strains separately. Among human strains, three additional VGs presented higher frequency in AIEC strains (*papGII/III, iss* and *vat*; *N* = 22) than in non-AIEC strains (*N* = 37). No differences between AIEC/non-AIEC were found in FimH variants. In contrast, the ChiA sequence of LF82 was shared with the 35.5% of AIEC studied (*N* = 31) and only with the 7.4% of non-AIEC strains (*N* = 27; *p* = 0.027). Binary logistic regression analysis, using as input variables all the VGs and antibiotic resistances tested, revealed that typifying *E. coli* isolates using *pic* gene and ampicillin resistance was useful to correctly classify strains according to the phenotype with a 75.5% of accuracy. Although there is not a molecular signature fully specific and sensitive to identify the AIEC pathotype, we propose two features easy to be tested that could assist in AIEC screening. Future work using additional strain collections would be required to assess the applicability of this method.

## Introduction

Inflammatory bowel disease (IBD) comprises a group of idiopathic conditions affecting the gastrointestinal tract. The two main types of IBD are Crohn’s disease (CD) and ulcerative colitis (UC), in which the intestinal microbiota has been proposed as a contributory agent (Sartor, 2006). An expansion of *Escherichia coli*, particularly the adherent-invasive *E. coli* (AIEC) pathotype, has been reported to occur in CD patients from several countries (Darfeuille-Michaud et al., 2004; Martin et al., 2004; Baumgart et al., 2007; Sasaki et al., 2007; Eaves-Pyles et al., 2008; Martinez-Medina et al., 2009a; Dogan et al., 2013; Céspedes et al., 2017). Today, the putative implication of AIEC in other gastrointestinal disorders (Darfeuille-Michaud et al., 2004; Martin et al., 2004; Sasaki et al., 2007; Schippa et al., 2009; Raso et al., 2011; Negroni et al., 2012; Prorok-Hamon et al., 2014; Raisch et al., 2014) and animal intestinal illnesses (Martinez-Medina et al., 2011; Dogan et al., 2014; Rahmouni et al., 2018) remains controversial.

The AIEC pathotype is defined as *E. coli* strains able to adhere to and invade intestinal epithelial cells (Boudeau et al., 1999), as well as to survive and replicate inside macrophages without inducing apoptosis but promoting the release of high levels of tumor necrosis factor alpha (Glasser et al., 2001). They lack common virulence factors of intestinal pathogenic *E. coli*, instead they present similar virulence traits to Extraintestinal Pathogenic *E. coli* (ExPEC) (Baumgart et al., 2007; Martinez-Medina et al., 2009b; Miquel et al., 2010; Nash et al., 2010).

The AIEC pathotype was described about 20 years ago in a patient with ileal CD (Boudeau et al., 1999) and since then substantial research has been conducted to elucidate the molecular mechanisms of AIEC virulence and its relation with the disease pathogenesis. Indeed, it has been demonstrated that AIEC benefit of CEACAM6 and CHI3L1 receptors, overexpressed in CD, to promote its adhesion and invasion to intestinal epithelial cells (IECs) located in the ileum via the FimH adhesin of the type-1 pili (Barnich et al., 2007; Carvalho et al., 2009) or to colonic IECs via the chitinase ChiA (Low et al., 2013), respectively. AIEC translocation may also occur through the M cells present in the Peyer’s patches by means of FimH and long polar fimbriae (LpfA; Chassaing et al., 2011). Translocated AIEC cells may survive macrophage engulfment and multiply inside mature phagolysosomes (Bringer et al., 2005, 2007), what implies continuous secretion of cytokines and chronic macrophage activation (Glasser et al., 2001). AIEC link with the disease is reinforced by its ability to stimulate granuloma formation *in vitro*, which is a common histopathological feature of CD (Meconi et al., 2007). Genetic defects (Lapaquette et al., 2012) and elevated expression of some microRNAs (Nguyen et al., 2014) related to impairment of autophagy in CD patients contribute to unrestrained AIEC intracellular replication and persistent infection.

Several approaches have been conducted in order to identify the genetic basis of AIEC phenotype so far, starting by a PCR detection of known virulence genes (Darfeuille-Michaud et al., 2004; Martinez-Medina et al., 2009a,b, 2011; Chassaing et al., 2011; Conte et al., 2014; Vazeille et al., 2016; Céspedes et al., 2017) and following with comparative genomics to find new genes and point mutations (Baumgart et al., 2007; Bronowski et al., 2008; Miquel et al., 2010; Nash et al., 2010; Dogan et al., 2014; Deshpande et al., 2015; Desilets et al., 2015; Zhang et al., 2015; O’Brien et al., 2016), but no AIEC specific and representative molecular marker has been identified yet.

Since AIEC identification still relays on phenotypic assays based on infected cell cultures, which are extremely time consuming and hard to standardize, the finding of molecular tools or rapid tests to easily identify the AIEC pathotype would definitely be of interest for scientists studying the epidemiology of the pathotype and clinicians that aim to detect what patients are colonized by AIEC to apply personalized treatments.

In this study, the prevalence and/or sequence variants in gene products of a number of virulence genes (VGs) as well as the antimicrobial resistance profile of AIEC and non-AIEC strains has been compared in order to look for signature traits that could assist in a rapid AIEC identification.

## Materials and Methods

### *E. coli* Collection

The *E. coli* collection used in this study was composed by three groups of strains: (I) strains previously isolated from the intestinal mucosa of CD patients and controls (C) under the approval of the Ethics Committee of Clinical Investigation of the Hospital Josep Trueta of Girona on May 22, 2006 (Martinez-Medina et al., 2009a), (II) strains previously isolated from animals suffering from enteritis under routine microbiological diagnostic procedures (Martinez-Medina et al., 2011), and (III) strains newly isolated from colorectal cancer (CRC) and UC patients (Supplementary Table 1). Biopsies from CRC and UC patients were taken from the ileum and/or colon with sterile forceps, immediately placed in sterile tubes without any buffer, and maintained at 4°C for *E. coli* isolation. The study protocols for CRC and UC strains were approved by the local Ethics Committees (CEIC-Institut d’Assistència Sanitària, in April 2009 and January 2012; and CEIC-Hospital Universitari de Girona Droctor Josep Trueta, in May 2006). All subjects gave written informed consent in accordance with the Declaration of Helsinki. Additionally, the AIEC reference strain LF82, which was a kind gift from Prof. Darfeuille-Michaud (Université d’Auvergne, France), was also included. Information about the strains examined in each section (VGs prevalence, FimH and ChiA sequence variants and antibiotic resistance) can be found in Table 1 and Supplementary Table 2. The phylogenetic distribution of the strains studied in each section according to pathotype and origin of isolation is presented in Supplementary Table 3.

**Table 1:** Strain collection studied in each section according to host, disease and pathotype.

Section	Host	Disease	AIEC	non-AIEC	B2-AIEC	B2- non-AIEC
Virulence	Human	CD	16	18	12	6
gene		C	6	19	3	8
prevalence						
	Animal	Enteritis	26	19	21	8

FimH and ChiA sequence variants	Human	CD	16	15	12	6
		C	6	12	3	7
		UC	7	0	ND	ND
		CRC	2	0	ND	ND

*pic*	Human	CD	16	15	12	6
prevalence and ampicillin resistance		C	6	12	3	7


*CD: Crohn’s disease; C: control; UC: ulcerative colitis; CRC: colorectal cancer. ND: not determined.*

### Virulence Genotyping by PCR

Fifty-four VGs from different groups, including adhesins, toxins, invasins, iron scavenging involved genes and genes involved in capsule formation and stress resistance, were amplified by PCR as defined previously (Martinez-Medina et al., 2011). In addition, *lpfA* genes have also been studied in human-isolated strains. PCR primers for *lpfA_141_* and *lpfA_154_* genes were extracted from Chassaing et al. (2011) and PCR conditions were applied as explained therein. All genetic elements studied (either genes or alleles) were referred as VGs in this work.

### Gene Sequencing and Sequence Analysis

For *fimH* gene, PCR primers and program conditions were applied as described elsewhere (Iebba et al., 2012). To sequence *chiA* gene, a set of four primers were designed in the present study. Two independent PCRs were performed in order to amplify the whole gene (2694 bp). The first PCR was carried with ChiA-84F (5′-TCATATTGAAGGGTTCTCG-3′) and ChiA1711R (5′-TCCAGTCAACAAAAACACGC-3′) leading to an amplicon of 1795 bp. The second PCR was carried with ChiA897F (5′-TAATAATGGCGGTGCTGTGA-3′) and ChiA+12R (5′-TCGCCAACACATTTATTGC-3′), what resulted in an amplicon of 1818 bp. Primers ChiA897F and ChiA1711R were used to sequence a fragment of approximately 550 bp in the middle of the gene in which previously described mutations were located. PCR products were purified by ExoSap (Thermo Fisher Scientific) following manufacturer’s instructions and sequenced by Sanger method (Macrogen, Netherlands). Sequences were cleaned and aligned with BioEdit software (Hall, 1999) using K-12 gene sequence as a reference (*fimH* gene ID: 948847; *chiA* gene ID: 947837) and uploaded in GenBank (MH730201 – MH730304). Nucleotide sequences were translated using EMBOSS Transeq (Rice et al., 2000). Reticulate trees were constructed with PopART software (Leigh and Bryant, 2015) using the median joining algorithm, considering only the variable DNA positions that caused non-synonymous amino acid changes.

### Adhesion and Invasion Assays

Adhesion and invasion assays were performed for isolates obtained from CRC and UC, whereas isolates from C, CD and animals were previously assessed (Martinez-Medina et al., 2009a, 2011; Camprubí-Font et al., 2018). Briefly, the Intestine-407 epithelial cell line (ATCC CCL-6) was used for the adhesion and invasion assays. Both assays were performed in triplicate as described previously (Boudeau et al., 1999). LF82 and K-12 strains have been used as positive and negative control, respectively. Adhesion values were indicated as number of bacteria per I-407 cell (bacteria/I-407 cell). Invasive ability was expressed as the percentage of the initial inoculum that became intracellular: I_INV (%) = (intracellular bacteria/4 × 10^6^ bacteria inoculated) × 100.

### Survival and Replication Within Macrophages

The replication capacity of AIEC isolated from CRC and UC, as well as, non-AIEC strains isolated from CD and C subjects was assessed in this study. The capacity of AIEC strains isolated from CD, C or animals were previously assessed (Martinez-Medina et al., 2009a, 2011). For survival and replication assays, the murine macrophage-like J774A.1 cell line (ATCC TIB-67) was used and assays were performed as depicted previously (Bringer et al., 2006). LF82 and K-12 strains have been used as positive and negative control, respectively. The results are expressed as the mean percentage of intracellular bacteria recovered at 1 and 24 h post-infection: I_REPL (%) = (CFU ml^-1^ at 24 h/CFU ml^-1^ at 1 h) × 100.

### Antibiotic Resistance

The collection of strains isolated from human was screened against 30 antimicrobial agents using the Vitek^®^2 system (Biomérieux), the Sensititre standard susceptibility plate COMPAN1F (TREK Diagnostic Systems) or the macrodilution test following the Clinical and Laboratory Standards Institute (CLSI) standards (Martinez-Medina et al., unpublished). Minimum Inhibitory Concentrations (MICs) were interpreted according to National Committee for Clinical Laboratory Standards (NCCLS) guidelines (Clinical and Laboratory Standards Institute, 2015).

### Statistical Analysis

The significance of frequency values, for prevalence of VGs was measured by Pearson’s χ^2^ test using SPSS 23.0 software according to phenotype and phylogroup. In terms of differences in the frequency of particular mutations in the FimH or ChiA protein sequence, Pearson’s χ^2^ test was used only for those variable positions harbored by more than three strains. For quantitative variables (adhesion and invasion index), the Mann–Whitney non-parametric test was applied. Binary Logistic Regression was employed to depict a predictive model to classify AIEC strains. All data about VGs prevalence, amino acid variants and antibiotic resistance were included in the model. In all cases, a *p*-value ≤ 0.05 was considered statistically significant.

## Results

### Virulence Gene Repertoires

#### Animal vs. Human *E. coli* Strains

**FIGURE 1:**
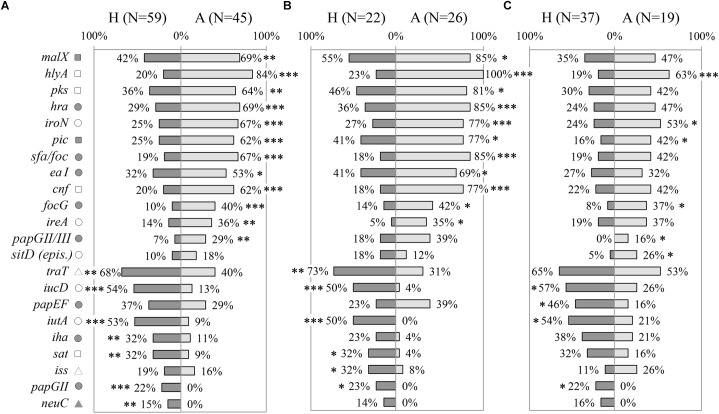
Distribution of virulence genes prevalence according to origin of isolation (H: strains isolated from humans; A: strains isolated from animals). **(A)** All *E. coli* strains. **(B)** Only AIEC strains. **(C)** Only non-AIEC strains. Numbers indicate gene prevalence in percentage in relation to the total of strains from each origin. Genes presenting statistically significant differences are depicted. Symbols indicate gene role in: adhesion (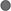), capsule formation (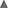), invasion (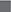), iron scavenging (o), resistance (Δ) and toxin (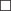). ^∗^*p*≤0.05; ^∗∗^*p*≤0.01; ^∗∗∗^*p*≤0.001.

Prevalence of 54 VGs were assessed in a collection of *E. coli* strains, including AIEC and non-AIEC, isolated from the intestine of both animals and humans (*N* = 104). Of those, 19 presented differential distribution according to host origin (Figure 1A, Supplementary Figure 1A and Supplementary Table 4). Twelve genes (*malX, hlya, pks, hra, iroN, pic, sfa/foc, eaI, cnf, focG, ireA* and *papGII/III*) were more frequent in animal-isolated strains (29–84%) than in human-isolated strains (7–42%; *p*≤0.025), and 7 genes (*traT, iucD, iutA, iha, sat, papGII* and *neuC*) were more prevalent in strains isolated from humans (present in 15–68% of total human strains) than in those from animals (0–40%; *p*≤0.010). Considering only those strains of the AIEC pathotype, 17 VGs were still associated with origin of isolation. Of those, 11 were more frequent in strains isolated from animals and 6 in human-strains (*p*≤0.046) (Figure 1B and Supplementary Table 4). In non-AIEC strains the prevalence of VGs was more similar when analyzing data by origin of isolation. In this case, 10 out of 54 genes were differentially distributed; six were overrepresented in animal strains and four in human strains (*p*≤0.038) (Figure 1C and Supplementary Table 4).

The distribution of virulence-associated genes was examined according to phylogroup. Considering the whole collection of strains, 55.6% (30 genes) of the VGs studied was associated with the phylogenetic origin of the strains (Supplementary Table 5). Most of the studied genes (29/30) were mainly related with B2 and/or D phylogroups, except for *csgA* gene, which was more frequent in A and B1 phylogroups. Of note, 17 of the 19 genes associated with either human or animal hosts were differentially distributed depending on the phylogenetic origin (Supplementary Table 5).

Considering that the distribution of phylogroups was different between animal and human strains (*p* < 0.001) (Supplementary Table 3), we selected the most abundant phylogroup (B2) to perform the comparisons, in order to avoid differences due to phylogenetic origin. Interestingly, genes previously associated with origin of isolation in the whole collection maintained its significance after selecting B2 strains only (Supplementary Table 4). Concerning AIEC and non-AIEC strains, 15/17 and 5/10 VGs respectively were still differentially distributed according to origin of isolation, when only B2 phylogroup strains were analyzed (Supplementary Table 4).

#### AIEC vs. Non-AIEC Strains

**FIGURE 2:**
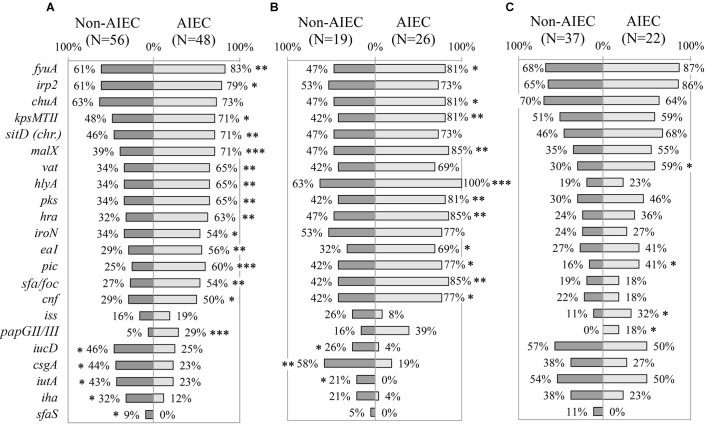
Distribution of virulence genes prevalence according to pathotype. **(A)** All *E. coli* strains. **(B)** Only animal-isolated strains. **(C)** Only human-isolated strains. Numbers indicate gene prevalence in percentage in relation to the total of strains from each pathotype. Genes presenting statistically significant differences were depicted. Symbols indicate gene role in: adhesion (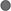), capsule formation (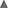), invasion (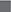), iron scavenging (o), resistance (Δ) and toxin (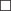). ^∗^*p*≤0.05; ^∗∗^*p*≤0.01; ^∗∗∗^*p*≤0.001.

The whole collection (*N* = 104) was evaluated to determine whether the prevalence of VGs was different between AIEC and non-AIEC strains. AIEC strains reported significantly higher prevalence than non-AIEC strains (*p*≤0.034) in: four genes related to adhesion capacity (*hra, eaI, sfa/foc* and *papGII/III*), four genes coding for toxins (*vat, hlyA, pks* and *cnf*), four genes linked with iron processes [*fyua, irp2, sitD (chr.)* and *iroN*], and three genes related to other functions [*kpsMTII* (capsule formation)*; malX* (metabolic processes) and*; pic* (invasiveness)] (Figure 2A, Supplementary Figure 1B, and Supplementary Table 6). In contrast, three genes involved in strain adhesiveness (*csgA*, *iha*, and *sfaS*) and two in iron processes (*iucD*, and *iutA*) were more frequent in non-AIEC than in AIEC strains (*p*≤0.026). Furthermore, higher adhesion for strains harboring *irp2, sitD (chr.), kpsMTII, vat* or *pic* (6.54 ± 7.75; 7.61 ± 7.84; 7.84 ± 8.63; 7.74 ± 8.11 and 8.32 ± 6.91 bacteria/cell VG-positive strains, respectively) was achieved in comparison with those that do not (2.64 ± 5.66; 3.13 ± 6.22; 3.08 ± 4.97; 3.69 ± 6.36 and 4.38 ± 7.39 bacteria/cell VG-negative strains, respectively; *p*≤0.046). In terms of invasion, *vat-*positive strains presented higher invasion values (0.31 ± 0.53%) than *vat*-negative strains (0.12 ± 0.21%) (*p* = 0.048). Additionally, the number of VGs present in each strain’s genotype was assessed according to pathotype. AIEC strains had from 4 to 30 VGs and non-AIEC carriage ranged from 4 to 33 VGs but, on average, AIEC strains tend to carry more VGs (18 ± 7 total number of genes) than non-AIEC strains (15 ± 8 total number of genes; *p* = 0.052). No significant differences were achieved probably due to high variation in the number of VGs carried between isolates.

All the genes reported to be differentially represented according to pathotype were also associated with phylogroup, with the exception of *sfaS* (Supplementary Table 5). Differential phylogroup distribution was reported between AIEC and non-AIEC strains studied in this section (*p* = 0.002), as non-AIEC strains were more predominant in A, B1 and D phylogroup while AIEC mainly constituted the B2 (Supplementary Table 3). Therefore, to prevent phylogroup as confounding factor, the analyses were performed only with B2 strains. Apart from three genes (*papGII/III, sfaS* and *pic*) that maintained its differential distribution between AIEC and non-AIEC strains, the others did not associate with pathotype (Supplementary Table 6).

To unveil possible differences in gene prevalence due to isolation origin, we further evaluated the 54 VGs in each group of strains (45 from animals and 59 from humans) (Figure 2B,C and Supplementary Table 6). Indeed, 13 out of the 20 genes found significant when analyzing all the strain collection, maintained the significance in strains isolated from animals but not in human strains. Only *pic* gene was more prevalent in AIEC strains irrespectively of strains’ host.

Among animal strains, *csgA, iucD*, and *iutA* genes were more prevalent in non-AIEC (21.10–57.90%) than in AIEC strains (0–19.20%; *p*≤0.040) while the remaining genes (*fyuA, kpsMTII, malX, hlyA, pks, hra, eaI, pic, sfa/foc* and *cnf*), plus an additional one (*chuA*) presented higher prevalence in AIEC (69.20–100%) than in non-AIEC strains (31.60–63.20%; *p*≤0.021) (Figure 2B and Supplementary Table 6). Additionally, phenotypic traits supported the difference in *hra* and *hlyA* gene prevalence between AIEC/non-AIEC strains. In this case, higher adhesion and invasion indices were obtained for those strains harboring *hra* (2.84 ± 3.86 bacteria/cell and 1.14 ± 2.15% for *hra*-positive strains; 2.28 ± 4.32 bacteria/cell and 0.66 ± 1.24% for *hra*-negative strains; *p* = 0.050 and *p* = 0.038, respectively) or *hlyA* (3.09 ± 4.18 bacteria/cell and 1.15 ± 2.05% for *hlyA*-positive strains; 0.34 ± 0.52 bacteria/cell and 0.13 ± 0.14% for *hlyA*-negative strains; *p* = 0.006 and *p* = 0.004, respectively). Among the animal strains, the phylogroup origin of AIEC and non-AIEC was different, being 72.4% of AIEC strains from B2 phylogroup while 68.8% of non-AIEC strains were from A phylogroup (*p* = 0.009) (Supplementary Table 3). If only B2 strains were selected, none of the 14 genes mentioned above (*csgA, iucD*, *iutA, hra, sfa/foc, fyuA, kpsMTII, cnf, hlyA, malX, pks, pic*, *eaI* and *chuA*) were found differentially distributed between pathogenic and commensal strains (Supplementary Table 6).

Regarding human-isolated strains, apart from *pic* gene, three additional genes (*papGII/III, iss*, and *vat*) reported significantly higher prevalence in AIEC (18.20–59.10%) than in non-AIEC strains (0–29.70%; *p* < 0.05) (Figure 2C and Supplementary Table 6). In addition, higher adhesion was reported for strains harboring *pic* (8.32 ± 6.91 bacteria/cell *pic*-positive strains and 4.38 ± 7.39 bacteria/cell *pic*-negative strains; *p* = 0.034) and higher adhesion (*p* = 0.043) and invasion (*p* = 0.048) values were obtained for *vat*-positive strains (7.74 ± 8.11 bacteria/cell and 0.31 ± 0.53%) in comparison with *vat*-negative strains (3.69 ± 6.36 bacteria/cell and 0.12 ± 0.21%). In this group of strains, similar phylogenetic distribution between AIEC and non-AIEC strains was observed (*p* = 0.072) (Supplementary Table 3). Nevertheless, 46.3% of the studied genes reported different prevalence regarding the phylogenetic origin (Supplementary Table 5). Thereby for following analyses only B2 strains were selected (Supplementary Table 6). In this case, none of the four genes (*papGII/III, iss, vat* and *pic*) associated with pathotype presented statistical differences, although a trend was noticeable for three of the cases (*papGII/III, pic* and *iss*) where the gene was more frequent in AIEC strains (*papGII/III:* 0% non-AIEC and 27% AIEC *p* = 0.057; *pic*: 29% non-AIEC and 60% AIEC *p* = 0.092; *iss:* 14% non-AIEC and 40% AIEC *p* = 0.129; and *vat:* 71% non-AIEC and 80% AIEC *p* = 0.458). In addition, the *astA* gene resulted to be more prevalent in non-AIEC (29%) than AIEC (0%) strains (*p* = 0.042).

Of note, genes previously found to be more frequent in AIEC than in non-AIEC strains from human (*lpfA_154_*, and *chuA*) (Dogan et al., 2014; Céspedes et al., 2017) reported similar percentage of PCR-positive AIEC/non-AIEC strains.

#### Crohn’s Disease vs. Controls

Differences in the VGs carriage were also reported between CD and C. In this case, four genes related with iron processes (*iha, iroN, iucD* and *iutA*) were more frequent in strains isolated from C than patients with CD (*iha*: 21% CD and 48% C, *p* = 0.026; *iroN*: 15% CD and 40% C, *p* = 0.029; *iucD*: 41% CD and 72% C, *p* = 0.018; *iutA*: 41% CD and 68% C, *p* = 0.037). On the other hand, a gene encoding for a meningitis-associated fimbria (*mat*) was more prevalent in CD-isolated strains (100%) than C (84%; *p* = 0.028). In addition, similar number of VGs was reported for CD strains (15 ± 7) in comparison to C strains (17 ± 8; *p* = 0.891). Similarly occurred when comparing VGs carriage in AIEC and non-AIEC strains according to disease origin (CD–AIEC 17.3 ± 6.3 vs. C–AIEC 14.7 ± 8.9, *p* = 0.169; CD–non-AIEC 13.1 ± 7.3 vs. C–non-AIEC 17.2 ± 7.2, *p* = 0.142).

### FimH and ChiA Amino Acid Substitutions

Since it has been suggested that differences regarding phenotype may rely on variations in the protein sequence, in this study alterations in FimH and ChiA have been explored. For this analysis, strains isolated from C, CD, UC and CRC were considered (*N* = 58; 31 AIEC and 27 non-AIEC strains).

**FIGURE 3:**
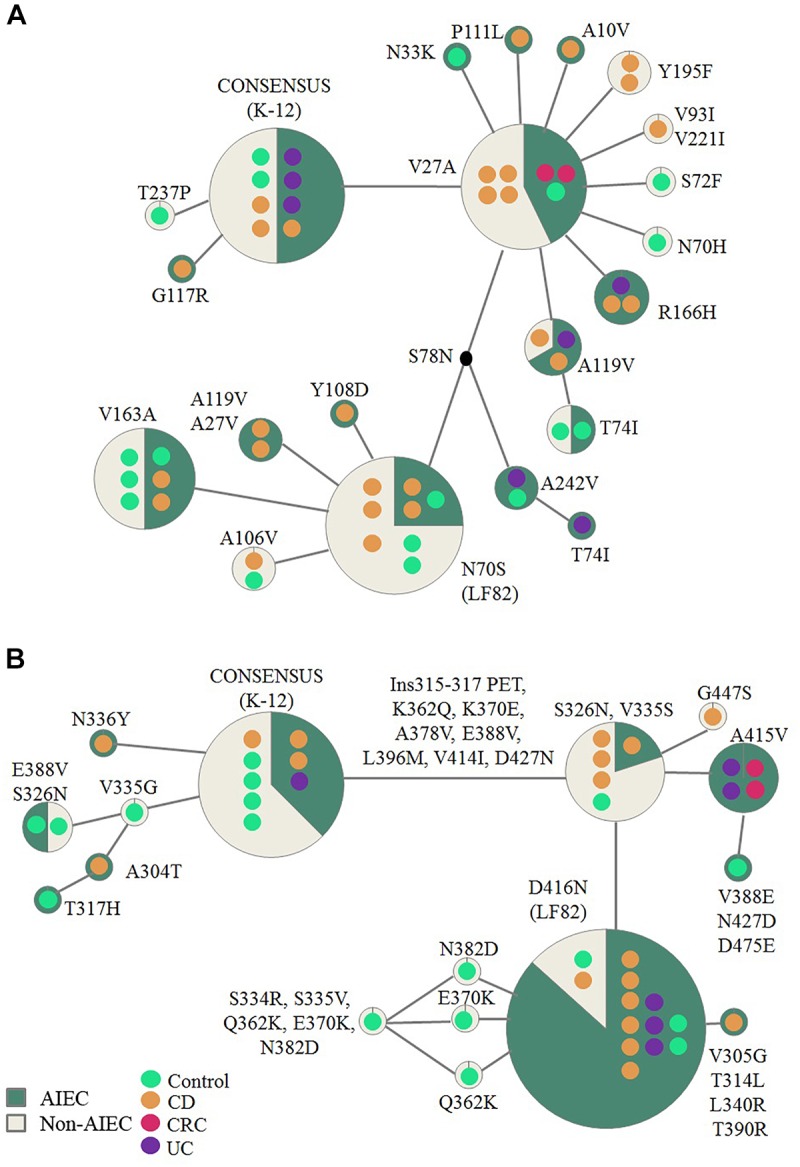
Reticulate tree representing FimH **(A)** and ChiA **(B)** variants. Each circle demonstrates the strains carrying specific mutations in the FimH/ChiA protein sequence. Number of strains is represented by the number of colored dots which also reflect origin of isolation. The amino acid changes indicated are derivatives of the consensus sequence (based on *E. coli* K-12 strain). AIEC pathotype proportion is indicated in green and non-AIEC in beige for each variant.

Fifty-four strains presented the *fimH* gene, representing 93.9% of AIEC and 92.6% of non-AIEC strains. As shown in Figure 3A and Supplementary Table 7, a total of 19 FimH amino acid substitutions were found among the strain collection which grouped the strains in 21 variants. There was no variant comprising uniquely or mainly AIEC strains. When comparing the sequence of AIEC/non-AIEC strains globally, both groups of strains presented on average two substitutions throughout the FimH sequence (AIEC: 2 ± 1; non-AIEC: 2 ± 1; *p* = 0.915). Individually, none of amino acid substitutions associated with the disease of isolation neither with AIEC phenotype. Only N70S and S78N were related to phylogenetic origin, as they were only found in C or CD-isolated strains from the B2 (69.2 and 73.1% of strains, respectively) and D (25.0 and 25.0% of strains, respectively) phylogroup (*p* < 0.001) (Table 2). Despite that, significant difference in terms of invasion index was achieved depending on the amino acid present in the 119 position but no divergence was found for the adhesion capacity. In this case, strains presenting the amino acid A (equal to K-12) had lower invasion values (0.223 ± 0.402% of intracellular bacteria/inoculum; *N* = 47) in comparison to strains with V (0.401 ± 0.477% of intracellular bacteria/inoculum; *N* = 7; *p* = 0.048).

**Table 2:** Frequency of amino acid substitutions for FimH and ChiA proteins in relation to pathotype and phylogroup.

	PATHOTYPE			PHYLOGROUP^a^				
FimH Position	AIEC (*N* = 29) (%)	non-AIEC (*N* = 25) (%)	*p*	A (*N* = 8) (%)	B1 (*N* = 6) (%)	B2 (*N* = 26) (%)	D (*N* = 4) (%)	*p*
V27A	75.9	84.0	NS	75.0	100	84.6	50.0	NS
N70S	31.0	40.0	NS	0.0	0	69.2	25.0	<0.001
S78N	41.4	40.0	NS	0	0	73.1	25.0	<0.001
A119V	17.2	8.0	NS	37.5	0	7.7	0	NS
V163A	10.3	12.0	NS	0	0	23.1	0	NS

**ChiA Position**	**AIEC** **(*N* = 25) (%)**	**non-AIEC** **(*N* = 17) (%)**	***p***	**A** **(*N* = 8) (%)**	**B1** **(*N* = 1) (%)**	**B2** **(*N* = 23) (%)**	**D** **(*N* = 1) (%)**	***p***

ins315_317 PET	68.0	64.7	NS	0	0	87.0	100	<0.001
S326N	76.0	70.6	NS	12.5	0	91.3	100	<0.001
V335G	12.0	11.8	NS	50.0	0	0	0	0.003
V335S	72.0	58.8	NS	0	0	87.0	100	<0.001
K362Q	72.0	52.9	NS	0	0	82.6	100	<0.001
K370E	72.0	52.9	NS	0	0	82.6	100	<0.001
A378V	72.0	64.7	NS	0	0	91.3	100	<0.001
E388V	72.0	70.6	NS	12.5	0	87.0	100	0.001
L396M	72.0	64.7	NS	0	0	91.3	100	<0.001
V414I	72.0	64.7	NS	0	0	91.3	100	<0.001
A415V	20.0	0	NS	0	0	4.3	0	NS
D416N	48.0	35.3	NS	0	0	65.2	0	0.008
D427N	64.0	64.7	NS	0	0	82.6	100	<0.001


*Only amino acid substitutions existent in more than three strains were examined. E. coli K-12 commensal strain was used as reference. ^a^Only strains isolated from Crohn’s disease or controls were considered. Atypical strain was discarded. NS: not significant.*

Regarding *chiA* gene, 86.2% AIEC strains (*N* = 31) and 63% non-AIEC strains (*N* = 27) presented this gene (*p* = 0.044). Twenty-four variable amino acid positions were found, assembling the strains in a total of 16 variants (Figure 3B and Supplementary Table 8). Again, similar protein sequence variants were reported among strains isolated from diverse groups of subjects (Figure 3). None of the mutations identified were associated with AIEC, neither the five mutations previously described (K362Q, K370E, A378V, E388V, V548E) (Low et al., 2013; Table 2). However, a subcluster of strains with *chiA* sequence identical to LF82 included a higher proportion of AIEC strains (85%) than non-AIEC strains (15%; *p* = 0.027). Nevertheless, this variant represented only the 35.5% of all AIEC strains and the 7.4% of total non-AIEC strains. Besides, the number of variable positions differed among pathotypes, being slightly higher for AIEC strains (10 ± 5) than in non-AIEC strains (8 ± 6; *p* = 0.038). Of note, most of the strains harboring an amino acid different from K-12 strain were from the B2 phylogroup, being V335G amino acid change an exception as it was only reported in A-phylogroup strains (Table 2).

### Test for Rapid AIEC Identification

To further establish a strategy that allows rapid identification of AIEC strains, we combined all the data of VGs carriage, amino acid variants and antibiotic resistance and performed Binary Logistic Regression to search for predictive features for AIEC screening (Table 3). In the present work, the combination of ampicillin resistance (Odds ratio = 5.244; 95% CI = 1.325–20.757) together with the prevalence of the *pic* gene (Odds ratio = 4.854; 95% CI = 1.140–20.638) uncovered a possible technique to identify AIEC strains, as it classifies strains according to the phenotype with a 75.5% of global success (P(AIEC) = –1.974+1.657 × ampicillin resistance + 1.579 × *pic* gene). For a given *E. coli* strain already isolated from human intestine that presents ampicillin resistance and harbors the *pic* gene, the probability to be AIEC would be of 87.81%. This probability is reduced to 59.76 and 57.87% if the strain has either ampicillin resistance or the *pic* gene, respectively, and it ends up to 22.07% if the strain is sensible to ampicillin and does not present the *pic* gene. Another combination resulted also significant (ampicillin resistance with *vat* gene prevalence). However, low sensitivity was achieved in this case (sensitivity 50%, specificity 77.8% and accuracy 65.3%).

**Table 3:** Binary logistic regression model evaluating the prevalence of the *pic* gene and ampicillin resistance as a putative method for AIEC identification.

	EQUATION VALUES			

	**B**	***p*-value**	**Odds ratio**	**95% CI**

***pic* gene**	1.579	0.033	4.851	1.140–20.638
**Ampicillin resistance**	1.657	0.018	5.244	1.325–20.757
Constant	-1.974	0.020	0.139	

	**PREDICTED**			

**OBSERVED**	**non-AIEC**	**AIEC**	**% Correct**	**Global%**

**Non-AIEC**	18	9	66.7	75.5
**AIEC**	3	19	86.4	

	**PROBABILITY TO BE AIEC**			

	***pic* positive (%)**	***pic* negative (%)**		

**Ampicillin Resistant**	87.81	59.76		
**Ampicillin Sensitive**	57.87	22.07		


## Discussion

The AIEC pathotype has been involved in CD, and our knowledge about its distribution in other intestinal or extraintestinal diseases as well as the reservoirs and transmission paths is scarce. One reason of that is due to the fact that AIEC identification is based on phenotypic traits undergoing cell-culture infection assays, which are extremely time consuming and hard to standardize. In this work we have deeply characterized genetically and phenotypically a collection of AIEC and non-AIEC strains isolated from the intestinal mucosa of human and animals with the aim to better define the characteristics of AIEC pathotype and to find putative genetic/phenotypic markers for its rapid identification.

In our collection, higher number of VGs were associated with animal than with human strains and although the phylogenetic origin determined VGs profiles, differences between human and animal strains were still evident when exclusively B2 strains were considered for comparison. This observation must be considered to further search for genetic traits associated with AIEC pathotype. The inclusion of animal strains in the study helped us to detect that the host origin of isolation needs to be carefully considered when drawing conclusions.

In the present work we have focused on strains isolated from humans. Four genes (*vat*, *pic*, *iss* and *papG*) differentially distributed between AIEC and non-AIEC strains have been identified. So far, there are limited studies in which the prevalence of these genes in AIEC strains has been investigated. Among these four genes, the vacuolating autotransporter toxin (*vat* gene) has been implicated in LF82 AIEC pathogenesis (Gibold et al., 2015). It encodes for an autotransporter toxin involved in the gut mucus degradation. We found higher frequency of *vat*-positive AIEC strains similar to two previous studies: Desilets et al. (2015) (9/13 AIEC and 0/6 non-AIEC) and Gibold et al. (2015) (32/75 AIEC and 10/70 non-AIEC). On the other hand, in our work, no differences in the prevalence of *vat* according to pathotype were described once only B2 strains were considered, as occurred in O’Brien et al. (2016). Nonetheless, higher adhesion and invasion values were reported for those strains harboring the *vat* gene, such as previously reported (Gibold et al., 2015). The *pic* gene also encodes for a protease with toxin autotransporter activity, so it could be also involved in AIEC pathogenesis. However, so far there have only been studies relating it with *Shigella flexneri* (Henderson et al., 1999), and strains from de Uropathogenic *E. coli*, Enteroaggregative *E. coli* and Enteroinvasive *E. coli* pathotypes (Boisen et al., 2009). To the best of our knowledge no study previously analyzed its presence in an AIEC collection. Herein, we reported occurrence of this gene in a subset of AIEC strains (41%) while it was less frequent in non-AIEC strains (16%). Moreover, higher adhesion values for *pic*-positive strains were found. This observation together with the fact that *pic* may contribute to intestinal colonization in mouse models for enteroaggregative *E. coli* (Boisen et al., 2009), suggest that the presence of *pic* might confer some bacterial virulence advantage. Isogenic mutants to confirm its implication in AIEC virulence are required. However, no differences between AIEC *pic*+ (60%) and non-AIEC *pic*+ (29%) strains was found once only B2-phylogroup strains were considered, a fact that may be attributable to the amount of strains analyzed. The *iss* (increased serum survival) gene encodes for a protein responsible for serum resistance in ExPEC, such as avian pathogenic *E. coli* strains (Johnson et al., 2008). In this case, Dogan et al. (2014) did not describe an association with AIEC, but probably differences in the phylogenetic origin of the strain collections may influence these results. Finally, the combination of alleles *papGII-III*, encoding for adhesins of the *E. coli* pilus P, have been found in a low percentage of human strains (12%). Nonetheless, this gene is involved in adhesion processes and has been suggested to contribute to the urosepsis’ pathogenesis (Féria et al., 2001). The prevalence of *papGII* has only been reported in AIEC strains isolated from CD pediatrics patients yet in a very low frequency compared with AIEC isolated from C (Conte et al., 2014).

Previous studies have reported differences in the prevalence of some genes according to pathotype (*pduC, chuA, lpfA, lpfA+gipA* and *vat*) (Dogan et al., 2014; Gibold et al., 2015; Vazeille et al., 2016; Céspedes et al., 2017). However, in our strain collection, similar *lpfA_154_* and *chuA* gene prevalence values were reported between AIEC and non-AIEC isolates. Bearing in mind that the VG carriage is deeply associated with the phylogenetic origin (Kotlowski et al., 2007), we suspect that these discrepancies may be explained due to the diversity of the strain collection used in each study. Therefore, our results confirm the high genetic variability of AIEC strains and suggest that many of the genetic features described to date are in fact related to phylogroup origin of the strains rather than to AIEC phenotype.

Results obtained on FimH, one of the most studied virulence factor in AIEC pathotype, are in line with previous data (Dreux et al., 2013; Desilets et al., 2015; O’Brien et al., 2016; Céspedes et al., 2017), since no differences in pathoadaptative mutations were specifically associated with AIEC pathotype. Besides, although Dreux et al. (2013) and Iebba et al. (2012) indicated that N70S and S78N FimH variants could confer increased strain’s capacity to adhere to the human receptor CEACAM6, no increased adhesion was observed for strains harboring these variants in our collection. Nonetheless, the strains with A119V mutation, a substitution previously reported to confer an advantage on adhesion (Iebba et al., 2012), presented higher invasion indices. Actually, N70S and S78N associated with B2 and D-phylogroup strains, as it has been determined in other groups of strains (Hommais et al., 2003; Miquel et al., 2010; Iebba et al., 2012; Dreux et al., 2013; Desilets et al., 2015). Finally, while G66S and V27A variants have been associated with CD origin of the strains and T74I, V163A and A242V variants with UC strains in a previous study (Iebba et al., 2012), no particular variants were associated with disease origin in the present work.

Up to our knowledge, this is the first study examining the sequence of ChiA in a large strain collection (*N* = 58). Until now, differences in ChiA sequence were only sought between LF82 and K-12 and five mutations (Q362K, E370K, V378A, V388E, and E548V) were described as required for the proper interaction between bacteria and epithelial cells (Low et al., 2013). Despite we found these mutations equally distributed between AIEC and non-AIEC strains and no significant differences neither in adhesiveness nor in invasiveness between variants, it is of note that the AIEC LF82 sequence variant was mainly shared among AIEC strains (being the 85% of strains with this sequence AIEC). Unfortunately this variant is not highly frequent amongst the whole AIEC collection, only the 35.5% of AIEC strains had this gene sequence variant, so we suggest this gene is not suitable for AIEC screening. Additional studies regarding the expression of *chiA* gene would be needed in order to decipher whether the strains harboring the same sequence express this gene differentially according to pathotype.

Scarce studies have evaluated the capacity of AIEC strains to resist the action of antibiotics (Subramanian et al., 2008; Dogan et al., 2013, 2018; Brown et al., 2015; Oberc et al., 2018) and no one has compared antibiotic resistance between AIEC and non-AIEC strains. In this work, we have combined this feature with VGs prevalence. Despite no specific and widely distributed AIEC characteristic has been found, in this work we show that the presence of *pic* gene and ampicillin resistance are two traits that could assist in AIEC screening since AmpR *pic* + *E. coli* strains have a probability of 82% to be AIEC. This could be of use as an initial method of screening of human *E. coli* isolates. The major problem is about false-positives, so AIEC predicted strains by this method should be further tested phenotypically. It is also necessary to test the specificity of the method using genetically close pathotypes such as Extraintestinal Pathogenic *E. coli* and to test the applicability in external strain collections isolated from different geographical locations.

To sum up, this data provide deepest knowledge about AIEC VGs sets, what has revealed four VGs that could be of relevance in AIEC pathogenicity. We reinforce the idea that no particular VG is related to AIEC phenotype. Despite diverse virulence factors could drive to the same phenotype, the presence of an AIEC-specific marker cannot be discarded. Differences in gene expression or point mutations of core genes may explain the genetic basis of AIEC pathotype. Noticeably, a novel strategy to assist in AIEC identification is proposed, yet further works confirming our results in additional strain collections are necessary.

## Author Contributions

MM-M designed the study and obtained funding. CC-F, MM-M, ML-S, and CE obtained the data. CC-F and MM-M performed the statistical analysis. CC-F drafted the manuscript. MM-M and ML-S revised the manuscript.

## Conflict of Interest Statement

The authors declare that the research was conducted in the absence of any commercial or financial relationships that could be construed as a potential conflict of interest.

## Supplementary Material

FIGURE S1 Venn diagram depicting the virulence genes statistically more prevalent in human or animal-isolated strains **(A)** or in AIEC or non-AIEC strains **(B)**. Genes no differentially distributed are: ^∗^*afa/draBC, astA, bmaE, chuA, csgA, cvaB, cvaC, eitA, eitC, etsB, etsC, fimC, fyuA, gafD, gimB, hlyF, ibeA, irp2, iss, kpsMTII, mat, nfaE, ompA, ompT, papC, papEF, papGI, papGIII, sfaS, sitA, sitD (chr.), sitD (epis.), tia, tsh*, and *vat*. ^#^*afa/draBC, astA, bmaE, chuA, cvaB, cvaC, eitA, eitC, etsB, etsC, fimC, focG, gafD, gimB, hlyF, ibeA, ireA, iss, mat, neuC, nfaE, ompA, ompT, papC, papEF, papGI, papGII, papGIII, sat, sitA, sitD (epis.), tia, traT*, and *tsh*.

TABLE S1 Information of the patients from whom the UC and CRC strains were isolated.

TABLE S2 Information about the strains analyzed. Host and disease of isolation, phylogroup origin, adhesion (ADH), invasion (INV) and intramacrophage replication (REPL) indices as well as country origin are indicated. VG prevalence, FimH and ChiA amino acid (aa) substitutions and AB resistance is depicted.

TABLE S3 Distribution of the phylogenetic origin of the strain according to pathotype **(A)** or origin of isolation **(B)** in each group of study. Values indicate the percentage of strains present in each condition.

TABLE S4 Prevalence of virulence-associated genes according to origin of isolation (all strains, AIEC or non-AIEC) considering all strains or B2 strains. Percentage values indicate, for each gene, the proportion of strains that obtained PCR positive amplification.

TABLE S5 Prevalence of virulence-associated genes according to phylogroup in *E. coli* strains isolated from humans and/or animals and AIEC/non-AIEC. Percentage values indicate, for each gene, the proportion of strains that obtained PCR positive amplification.

TABLE S6 Prevalence of virulence-associated genes according to pathotype in all or B2 *E. coli* strains isolated from humans and/or animals. Percentage values indicate, for each gene, the proportion of strains that obtained PCR positive amplification.

TABLE S7 Distribution of FimH amino acid substitutions among the strain collection. Numbers of strains in each variant are indicated.

TABLE S8 Distribution of ChiA amino acid substitutions among the strain collection. Numbers of strains in each variant are indicated.
